# RND2 attenuates apoptosis and autophagy in glioblastoma cells by targeting the p38 MAPK signalling pathway

**DOI:** 10.1186/s13046-020-01671-2

**Published:** 2020-08-31

**Authors:** Yang Xu, Qian Sun, Fan’en Yuan, Huimin Dong, Huikai Zhang, Rongxin Geng, Yangzhi Qi, Xiaoxing Xiong, Qianxue Chen, Baohui Liu

**Affiliations:** 1 Department of Neurosurgery, Renmin Hospital of Wuhan University, 238 Jiefang Street, Wuhan, 430060 Hubei China; 2 Central laboratory, Renmin Hospital of Wuhan University, Wuhan, 430060 Hubei China; 3 Department of Neurology, Renmin Hospital of Wuhan University, Wuhan, 430060 Hubei China

**Keywords:** RND2, p38 MAPK, Apoptosis, Autophagy, Glioblastoma

## Abstract

**Background:**

Inhibition of p38 MAPK signalling leads to glioblastoma multiform (GBM) tumourigenesis. Nevertheless, the molecular mechanism that induces p38 MAPK signalling pathway silencing during GBM genesis has yet to be determined. Identifying new factors that can regulate p38 MAPK signalling is important for tumour treatment.

**Methods:**

Flow cytometry, TUNEL assays, immunofluorescence, JC-1 assays, and western blot analyses were used to detect the apoptosis of GBM cells. The specific methods used to detect autophagy levels in GBM cells were western blot analysis, LC3B protein immunofluorescence, LC3B puncta assays and transmission electron microscopy. The functions of these critical molecules were further confirmed in vivo by intracranial xenografts in nude mice. Tumour tissue samples and clinical information were used to identify the correlation between RND2 and p62 and LC3B expression, survival time of patients, and tumour volumes in clinical patients.

**Results:**

By summarizing data from the TCGA database, we found that expression of the small GTPase RND2 was significantly increased in human glioblastomas. Our study demonstrated that RND2 functions as an endogenous repressor of the p38 MAPK phosphorylation complex. RND2 physically interacted with p38 and decreased p38 phosphorylation, thereby inhibiting p38 MAPK signalling activities. The forced expression of RND2 repressed p38 MAPK signalling, which inhibited glioblastoma cell autophagy and apoptosis in vitro and induced tumour growth in the xenografted mice in vivo. By contrast, the downregulation of RND2 enhanced p38 MAPK signalling activities and promoted glioma cell autophagy and apoptosis. The inhibition of p38 phosphorylation abolished RND2 deficiency-mediated GBM cell autophagy and apoptosis. Most importantly, our study found that RND2 expression was inversely correlated with patient survival time and was positively correlated with tumour size.

**Conclusions:**

Our findings revealed a new function for RND2 in GBM cell death and offered mechanistic insights into the inhibitory effects of RND2 with regard to the regulation of p38 MAPK activation.

## Background

As the most frequently seen primary intracranial tumour, glioma has the highest fatality rate of tumours in the central nervous system of adults, and half of cases are glioblastomas [1]. It is difficult to remove the tumour completely. To make things worse, unclear boundaries with normal tissue easily lead to tumour recurrences [2]. According to gene expression profiling studies, GBM can be categorized into three transcriptionally defined and clinically related subtypes: proneural (PN), mesenchymal (MES) and classical (CL) [3]. Despite the fact that standard therapies including surgery, chemotherapy with TMZ and radiotherapy have developed rapidly, the one-year survival rate remains only 40.6%, and the five-year survival rate is only 5.6% [1]. The resistance to cell death is one of the hallmarks of GBM cells [4], therefore, it is crucial to clarify the specific molecular mechanisms of this phenomenon to identify a novel therapeutic target for GBM.

p38 mitogen-activated protein kinases are a class of mitogen-activated protein kinases (MAPKs) that are responsive to various stresses and are involved in different cell processes, including cell death [5, 6]. p38 MAPK signalling plays an important role in GBM; p38 MAPK signalling pathway activation is achieved via a phosphorylation cascade [7], and phosphorylated p38 can regulate key proteins in autophagy and apoptosis, leading to cell death. As a result, p38 is considered an inducer of apoptosis in GBM [8]. However, the regulation of p38 MAPK signalling in GBM still remains unclear. Hence, understanding the regulation of p38 MAPK may provide a new insight into the mechanisms of cell death and potential strategies for GBM therapy.

As an atypical member of the Rho GTPase family, RND2 does not have detectable GTPase activity. The most distinctive function of RND2 is its inhibitory effect on Rho kinase-mediated biological functions, including actin cytoskeleton formation and phosphorylation of myosin light chain phosphatase [9]. Recent studies have also pointed out that RND2 is a key regulator of neuronal movement in the development of the brain and is also essential in regulating the actin cytoskeleton of cells [10, 11]. Relatedly, as a novel and specific effector of Rnd2 GTPase, Rapostlin induces neurite branching [12]. However, the pathological role of RND2 in human GBM progression has not been investigated and associated animal studies need to be explored.

Here, we have provided evidence showing that RND2 is an oncogene in human glioblastomas. RND2 expression levels were significantly upregulated in human glioblastomas and suggest a poor prognosis in patients. Additionally, RND2 physically interacted with p38 and decreased p38 phosphorylation, which reduces expression of its downstream substrates, LC3B, Beclin-1, cleaved-caspase3 [13] and induces p62 [14, 15]. In conclusion, our findings revealed a new function for RND2 in GBM cell death and provide new insight into the inhibitory effect of RND2 on regulatory mechanisms of p38/MAPK activation.

## Methods and materials

### Bioinformation analysis

The expression profiles of RND2 in different human cancers were downloaded from the TCGA database, while the profiles in normal human tissues were based on information from the HPA database (https://www.proteinatlas.org). RND2 expression profiles in different glioma subtypes were analysed based on the GlioVis portal (https://gliovis.bioinfo.cnio.es) [16].

### Human GBM and control brain tissues

Human control brain tissues and GBM tissues were acquired from the Department of Neurosurgery, Renmin Hospital of Wuhan University. GBM tissues were sampled during surgeries and stored at − 80 °C. Control brain tissues were collected from patients during emergency surgeries for traumatic brain injury. The procurement and use of tissue in this study was approved by the Renmin Hospital of Wuhan University’s Institutional Ethics Committee of the Faculty of Medicine (approval number: 2012LKSZ (010) H). The histological diagnosis of glioma was confirmed by the pathologists of the Department of Pathology at the Renmin Hospital of Wuhan University. All tumour samples were subjected to pathological examination and related molecular testing (MGMT, 1p19q, and IDH1/IDH2), and all were defined according to the 2016 WHO classification [17]. All clinical information for the patients is listed and presented in Supplemental Table S1.

### Cell culture

The human renal epithelial cell line (293 T) and human GBM cell lines (U87 and U251) were from the Cell Bank of the Shanghai Institute of Biochemistry and Cell Biology (Shanghai, China). Information detailing the U251 and U87 cell lines, the generation of the U87 stable cell line, and cell culture methods was described in our previous study [18]. Cells were cultivated in DMEM along with 1% penicillin/streptomycin and 10% foetal bovine serum, and the incubating temperature was 37 °C, with 5% CO2. The STR Authentication is listed in the supplemental materials.

### Reagents and antibodies

Antibodies used in these experiments included the following: anti-RND2 (13844–1-AP, Proteintech, USA), anti-Rho7/Rnd2 (GXT56070, GeneTex, USA), anti-p-p38 (#4511, Cell Signaling Technology, USA), anti-p38 (#9212, Cell Signaling Technology, USA), anti-cleaved-caspase3 (ab32042, Abcam, UK), anti-caspase3 (NB100-56708SS, Novus, USA), anti-BAX (50599–2-Ig, Proteintech, USA), anti-GAPDH (#5174, Cell Signaling Technology), anti-P62 (M162–3, Medical Biological Laboratories, Japan), anti-Beclin1 (11306–1-AP, Proteintech, USA), anti-LC3B (GB11124, Servicebio, China), anti-DYKDDDK/Flag-tag (ANT102, Antgene, China), and anti-His-tag (D291–3, Medical Biological Laboratories, Japan). The autophagy inhibitor wortmannin (3-MA) and the autophagy activator rapamycin (Sirolimus) (S1039, USA) were purchased from Selleck (S2758, USA).

### Quantitative real-time PCR (qPCR) and RNA extraction

The extraction of total RNA from tissues and cells was carried out using the Trizol reagent (Invitrogen, USA). For the reverse transcription of RNA, the PrimeScript RT Reagent Kit (RR047A, Takara, Japan) was used to synthesize cDNA. Using SYBR Premix Ex Taq II (RR820A, Takara), we performed qPCR to detect mRNA levels following the specifications provided by the manufacturers. qPCR was performed on a 2.1 Real-Time PCR System using Bio-Rad CFX Manager (Bio-Rad, USA). The relative Ct method was adopted to compare the data of the experimental group and the control group, and GAPDH was set as internal control. The primer sequences are listed in Supplemental Table S3. The clinical information of the GBM patients who provided samples is listed in Supplemental Table S2.

### shRNA transfection

Specific shRNA targeting of human RND2 (shRND2) and negative control shRNA (shNC) were purchased from RiboBio Corporation (Guangzhou, China). Referring to the specifications, Lipofectamine 3000 transfection reagent (L3000015, Thermo Fisher Scientific) was used in the transfection. The sequences of the different shRND2 constructs are provided in Supplemental Table S3.

### DNA construction and transfection

RND2 cDNA was subcloned with a Flag tag (Flag-RND2) into the pcDNA3.1 vector. Full-length p38 cDNA was subcloned with a 6x His tag (His-p38) into the pcDNA3.1 vector. Transfections were carried out with the help of the transfection reagent Lipofectamine 3000 (L3000015, Thermo Fisher Scientific) in accordance with the specifications.

### Flow Cytometric analysis

An Annexin V-PE/7-AAD kit (Becton Dickinson, New Jersey, USA) was used to measure the apoptosis rate of GBM cells. GBM cells were collected and then washed with PBS three times. Then, samples were stained with Annexin V-PE/7-AAD for 15 min in the dark. One hour after staining, the specific apoptosis of GBM cells was analysed using a FACSCalibur flow cytometer (Becton Dickinson). Negative staining for both 7-AAD and Annexin V-PE suggested that the cells were still viable with no apoptosis. Cells in the early stage of apoptosis were positive for Annexin V-PE and negative for 7-AAD. Positive staining for both 7-AAD and Annexin V-PE meant that the cells were in the late stage of apoptosis, or were already dead. To calculate the total apoptosis rate and carry out statistical analysis, the sum of the upper and lower right quadrants was calculated.

### Mitochondrial membrane potential (ΔΨm) assay

For early stage apoptosis, the collapse of Δψm function is a hallmark event. Δψm variations were detected by capturing the images of cells after JC-1 staining (Yeasen, Shanghai, China) using an Olympus BX51 microscope (Olympus, Japan) operated followed the manufacturer’s specifications. We also recorded the ratio of aggregated JC-1 (red fluorescence) to monomeric JC-1 (green fluorescence). ImageJ software was used to detect the fluorescence intensity. A drop in the red/green fluorescence intensity ratio indicated the loss of ΔΨm.

### TUNEL assay

A feature of apoptotic cells is the fragmentation of DNA, which can be measured using a TUNEL kit. We followed the protocol offered by the manufacturer of the TUNEL kit (Roche Diagnostics, Mannheim, Germany). Images of stained cells were collected using the Olympus BX51 microscope (Olympus, Japan). ImageJ software was used to count TUNEL positive cells.

### Western blotting

Cells or tissues were lysed in RIPA buffer with protease and phosphatase inhibitors (cocktails from Roche and PMSF from Beyotime) for 30 min at 4 °C. The protein concentration was detected using a BCA kit (Biosharp, China). Proteins were separated by SDS-PAGE and then transferred onto a PVDF membrane, which was incubated with primary antibodies (including anti-GAPDH, anti-Flag, anti-β-actin, anti-p-p38, anti-Rnd2, anti-p38, anti-His, anti- p62, anti-caspase3, anti-LC3B, anti-Beclin1, anti-BAX, and anti-cleaved-caspase3) overnight and secondary antibodies for an hour. The proteins were delineated with a LI-COR Odyssey Infrared imaging system (LI-COR Bioscience, USA). ImageJ software was used to detect the grey value of the blots. The relative protein quantity was normalized to GAPDH.

### Immunoprecipitation assays

Cell lines U87 and U251 were co-transfected with the Flag-Rnd2 (Miaoling Biology, China) and His-p38 (Miaoling Biology, China) plasmids. Cells were lysed in IP buffer containing 1% NP-40, 50 mM NaF, 2 mM Na3VO4, 4 mM Na pyrophosphate and protease inhibitors 48 h after transfection. A total of 3 μg of antibodies (anti-Flag, anti-His or IgG; Beyotime) were added to the cell lysates, and the samples were incubated with 30 μL Protein A/G (Santa Cruz Biotechnology) at 4 °C overnight. The precipitates were washed 5 times or more with IP buffer and were boiled for 5 min in 40 μL 1.5x loading buffer (Beyotime), followed by western blot.

### Immunofluorescence assays

Cells were fixed in 4% paraformaldehyde for 15 min, treated with 0.1% Triton-X for 10 min and blocked with 1% BSA for 1 h. The samples were then incubated with primary antibodies (anti-p38, anti-Rnd2, anti-pp38, anti-Lc3B, anti-Cleaved-Caspase3) overnight, followed by Alexa Fluor-labelled secondary antibodies (Antgene, China). The nuclei were stained by DAPI (ANT046, Antgene, China). The Olympus BX51 microscope (Olympus) and a FV1200 confocal microscope (Olympus) were used to take pictures. ImageJ software was used to count positive cells.

### Immunohistochemistry

The tissues were embedded in paraffin after being fixed in 4% paraformaldehyde and cut into slices. After being hydrated, the slices were treated with 3% H2O2 for 10 min and blocked with 1% BSA for 1 h. The samples were then incubated with primary antibodies (anti-LC3B, anti-cleaved-Caspase, anti-p-p38, anti-BAX, anti-Rnd2) overnight, followed by HRP-labelled secondary antibodies (Servicebio, China). DAB (Servicebio, China) was used for dyeing and haematoxylin was used to stain the nuclei. Pictures were taken with the Olympus BX51 microscope (Olympus). A semiquantitative score was applied to describe the distribution and intensity of RND2 staining (0 = negative, 1 = weak, 2 = moderate, 3 = strong, and 4 = strong and widely distributed).

### Assay of green fluorescent protein-LC3 Puncta

RND2 plasmids were transfected into GBM cells that stably expressed green fluorescent protein (GFP)-LC3. After 2 days, transfected cells were fixed with 4% paraformaldehyde, and then a confocal laser scanning microscope (Olympus, Japan) was used to visualize GFP-LC3 puncta in the cells. The number of GFP-LC3 puncta was calculated from at least 100 cells.

### Transmission Electron microscopy (TEM)

Cells transfected with CTRL or RND2 plasmids were fixed with an electron fixation solution containing 2.5% glutaraldehyde. The cells were then post-fixed in 1% osmic acid. Next, a graded series of ethanol was used to dehydrate the specimens. They were then placed in capsules contained embedding medium and heated at 70 °C for approximately 9 h. The specimen sections were stained by uranyl acetate and alkaline lead citrate. Finally, the stained sections were observed using a TEM (Hitachi HT7700, Tokyo, Japan).

### Intracranial Xenograft model

PBS was used to suspend U87-MG cells, which stably expressed lentivirus RND2 or CTRL plasmids, at a concentration of 1 × 10^5^ cells/μL, and the cells were then injected into the right striatum of 6 week old Balb/c nude mice by stereotactic implantation; a blank control group that only received PBS was included. For the analysis of survival, the mice were under periodic monitoring and they were sacrificed when serious neurological symptoms appeared and/or an evident loss of weight (more than 20% of their body weight) occurred. We removed and weighed the whole mouse brains. The values (weight of control group/RND2 overexpression group - blank control group) were calculated and statistically analysed. All samples were then fixed in 4% paraformaldehyde. The brains were kept for further analysis and were embedded in paraffin. The Institutional Animal Care and Use Committee of the Renmin Hospital of Wuhan University approved all animal experiments mentioned above.

### Statistical analysis

All experiments were done in triplicate and were replicated at least once. All data are expressed as the means ± standard deviations. Statistical analyses were carried out with GraphPad Prism 7 and SPSS version 19.0. Unpaired Student t tests were used in the comparison of means between two groups. *p* values less than 0.05 were considered to be statistically significant. One-way analysis of variance (ANOVA) was performed to determine the differences between groups. When the analysis showed significance, post hoc testing that targeted the differences between groups was carried out using the Student-Newman-Keuls test. The Pearson correlation coefficient was used to analyse the correlation between RND2 and other genes. *P < 0.05, **P < 0.01, ***P < 0.001 was considered significant statistics.

## Results

### Rnd2 was upregulated in human GBM

**Fig. 1:**
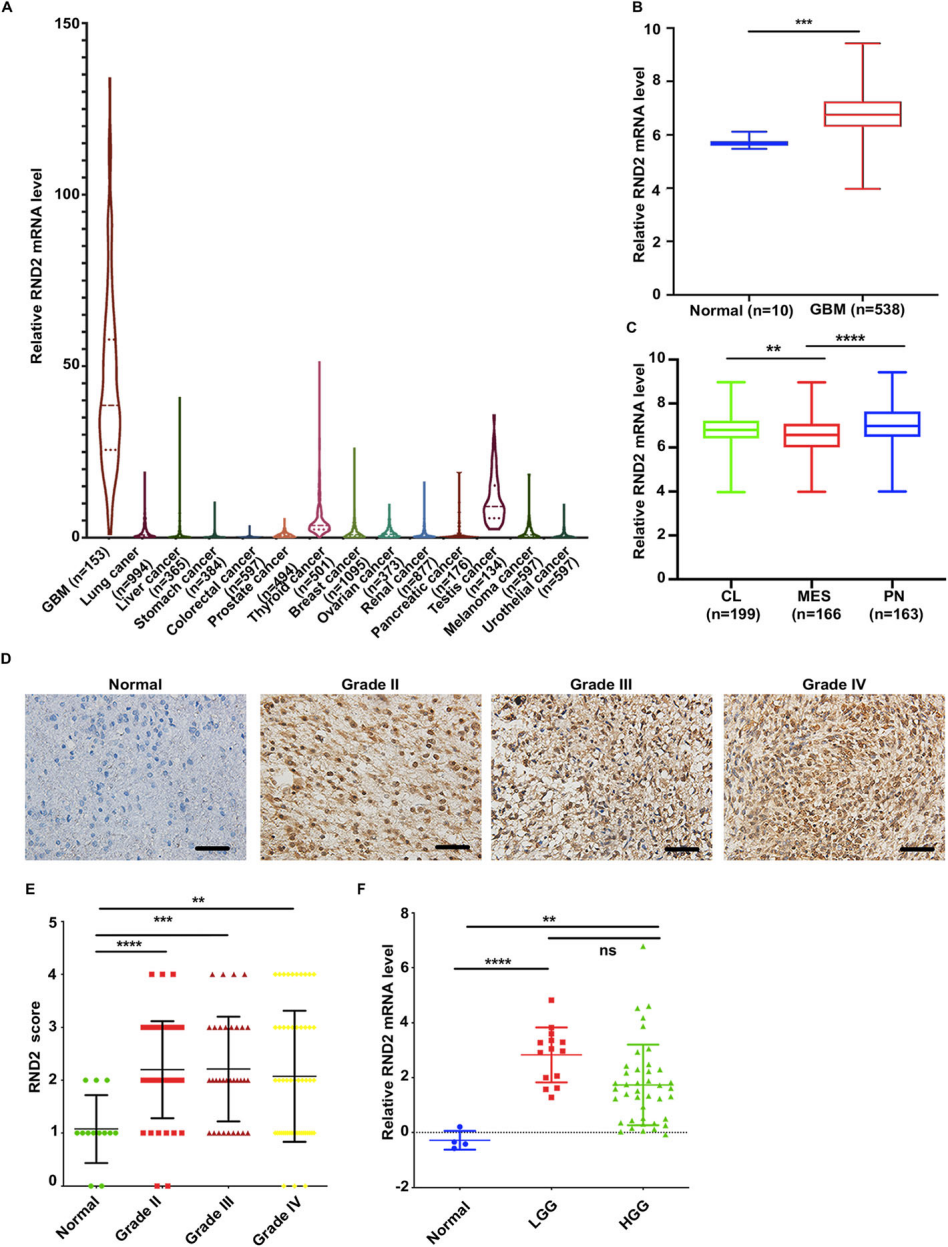
Rnd2 is upregulated in human GBM. **a** Expression of RND2 in 14 categories of human cancers according to the TCGA database. **b** Comparison of RND2 expression levels between GBM and normal brain tissues. **c** Comparison of RND2 expression levels between GBM MES, PN, or CL subtypes. Boxplots indicate the median quartiles, with whiskers extending the minimum and maximum range. **d** Representative IHC staining images for RND2 in clinical tissues. Grade II, grade III and grade IV indicate the pathologic grades of the glioma samples. Scale bars, 20 μm. **e** IHC score of RND2 in clinical tissues. The IHC scores were graded as 0, 1, 2, 3 and 4. Non-tumour tissue, *n* = 14; WHO II, *n* = 31; WHO III, *n* = 41; and WHO IV, *n* = 54. **P < 0.01, ***P < 0.001, ****P < 0.0001. **f** Real-time qPCR indicated significantly higher levels of RND2 mRNA in GBM (n = 41) tissues and LGG (n = 14) compared to non-tumour (n = 4) tissues. RND2 mRNA expression was normalized to GAPDH using the 2-ΔΔCt method. **P < 0.01, ****P < 0.0001

First, using the public TCGA database, we studied the expression profile of RND2 in diverse human cancers (http://cancergenome.nih.gov/). The results showed that among 14 categories of human cancer, RND2 expression was evidently higher in glioblastomas (Fig. 1a). We also analysed RND2 expression profiles in diversified human tissues based on the Human Protein Atlas database (http://www.proteinatlas.org/). We found that RND2 expression was obviously higher in the brain compared to other tissues (Fig. S1A). Furthermore, RND2 expression was significantly upregulated in GBM compared with normal brain tissue according to TCGA database (Fig. 1b). Consistent with these findings, we carried out western blot and RT-PCR assays to analyse the protein and the mRNA levels of RND2 from different organs of C57 mice and found that RND2 was highly expressed in the brain (Fig. S1B, C). We also found that RND2 expressed in human brain under normal condition (Fig. S1D, E). Further, we measured the expression of RND2 in the three sub-classes of glioblastomas and found that, compared with the PN or CL GBM subtypes, RND2 expression was significantly lower in MES GBM (Fig. 1c). To determine whether the levels of RND2 protein were elevated or not in clinical samples, we analysed the expression of RND2 in human glioblastoma samples, including 14 normal brain tissue samples, 31 WHO grade II gliomas, 41 WHO grade III gliomas and 52 glioblastomas, and we found that RND2 expression was significantly higher in gliomas compared with that of normal brain tissues (Fig. 1d, e). Information about all of the patients is listed in Supplementary Table S1. Furthermore, we found that the level of RND2 mRNA was significantly increased in gliomas, regardless of whether they were low-grade or high-grade gliomas (Fig. 1f). All of these data suggested that RND2 was upregulated and acted as a potential oncogene in GBM, hence, we further explored the function of RND2 in GBM.

### RND2 knockdown induced GBM cell apoptosis in vitro

**Fig. 2:**
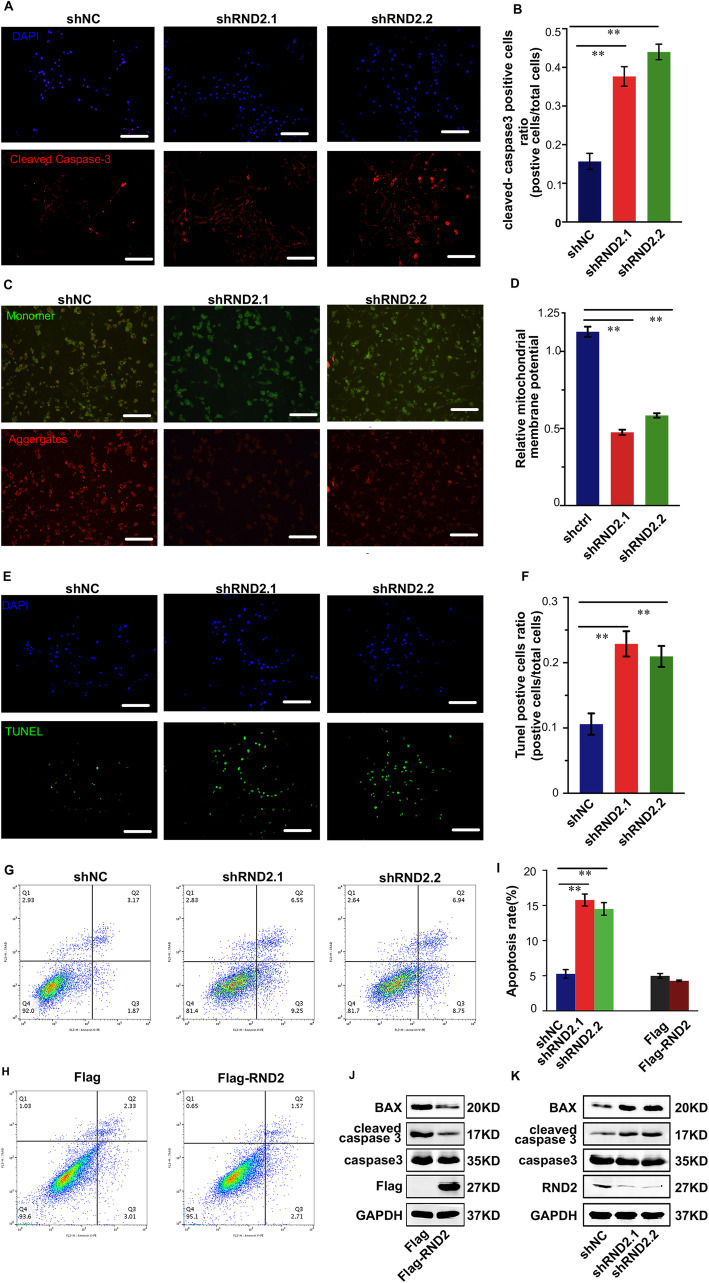
RND2 knockdown induced GBM cell apoptosis in vitro. **a-b** Effects of RND2 knockdown on cleaved Caspase-3 expression in U87 cells according to immunofluorescence. Scale bars, 100 μm. **P < 0.01. **c-d** Effects of RND2 knockdown on the ΔΨm in U87 cells according to JC-1 staining. A decrease in the ratio of red (aggregates)/green (monomers) fluorescence intensity indicates the loss of ΔΨm. Scale bars, 50 μm. **P < 0.01. **e-f** U87 cell death was detected by TUNEL staining. Scale bars, 50 μm. Statistical analysis of the positive rate is showed. **P < 0.01. **g-i** U87 cells were transfected with negative control shRNA (shNC) or shRNAs against RND2 (shRND2–1 and shRND2–2), followed by Annexin V-PE/7-AAD staining and flow cytometric analysis. Cell apoptosis was calculated by FACS. **P < 0.01. **j-k** Effects of RND2 overexpression and knockdown on the levels of apoptosis-related proteins in GBM cells. shNC: negative control shRNA; shRND2–1 and shRND2–2: two shRNAs against RND2; Flag: the control group; Flag-RND2: RND2 overexpression group. All bar plot data are the means ± SD. The data and graphs are representative of three independent experiments with similar results

We conducted functional studies to detect apoptosis in U87 and U251 cells to track the function of RND2 in GBM. Different shRNAs (shRND2–1 and shRND2–2) targeting RND2 were designed and their knockdown efficacies were ensured by western blot (Fig. 2k and Fig. S2C). First, to evaluate cell death, we performed immunofluorescence and immunoblotting for cleaved-caspase3 and found that the RND2 knockdown groups expressed a significantly higher level of cleaved-caspase3 in contrast to the control group in both U87 cells and U251 cells (Fig. 2a, b, k and Fig. S2C). It is known that one of the hallmark events in the early stage of apoptosis is the loss of ΔΨm [19]. JC-1 staining demonstrated that RND2 knockdown induced the loss of ΔΨm in GBM cells (Fig. 2c, d). Furthermore, we performed TUNEL staining and the results demonstrated that the number of TUNEL-positive cells was significantly elevated in the RND2 knockdown groups (Fig. 2e, f). Annexin V-PE/7-AAD staining, which was detected by flow cytometry, showed that RND2 knockdown increased apoptosis in both U87 and U251 cells (Fig. 2g, i and Fig. S2A, B). When RND2 was overexpressed in U87 cells, the apoptosis rate did not decrease significantly, which may be due to the low level of apoptosis in these cells under normal conditions (Fig. 2h, i).

To further support these data, immunoblotting was also used to examine the expression of the BCL-2-associated X protein (BAX) when RND2 was up- or downregulated in U87 and U251 cells. The results showed that the expression of BAX was decreased when RND2 was overexpressed in U87 and U251 cells, and reduced levels of RND2 resulted in the opposite effects (Fig. 2j, k and Fig. S2C).

Collectively, all of these data suggested that apoptosis of glioblastoma cells was negatively regulated by RND2 expression.

### RND2 overexpression reduced GBM cell autophagy in vitro

**Fig. 3:**
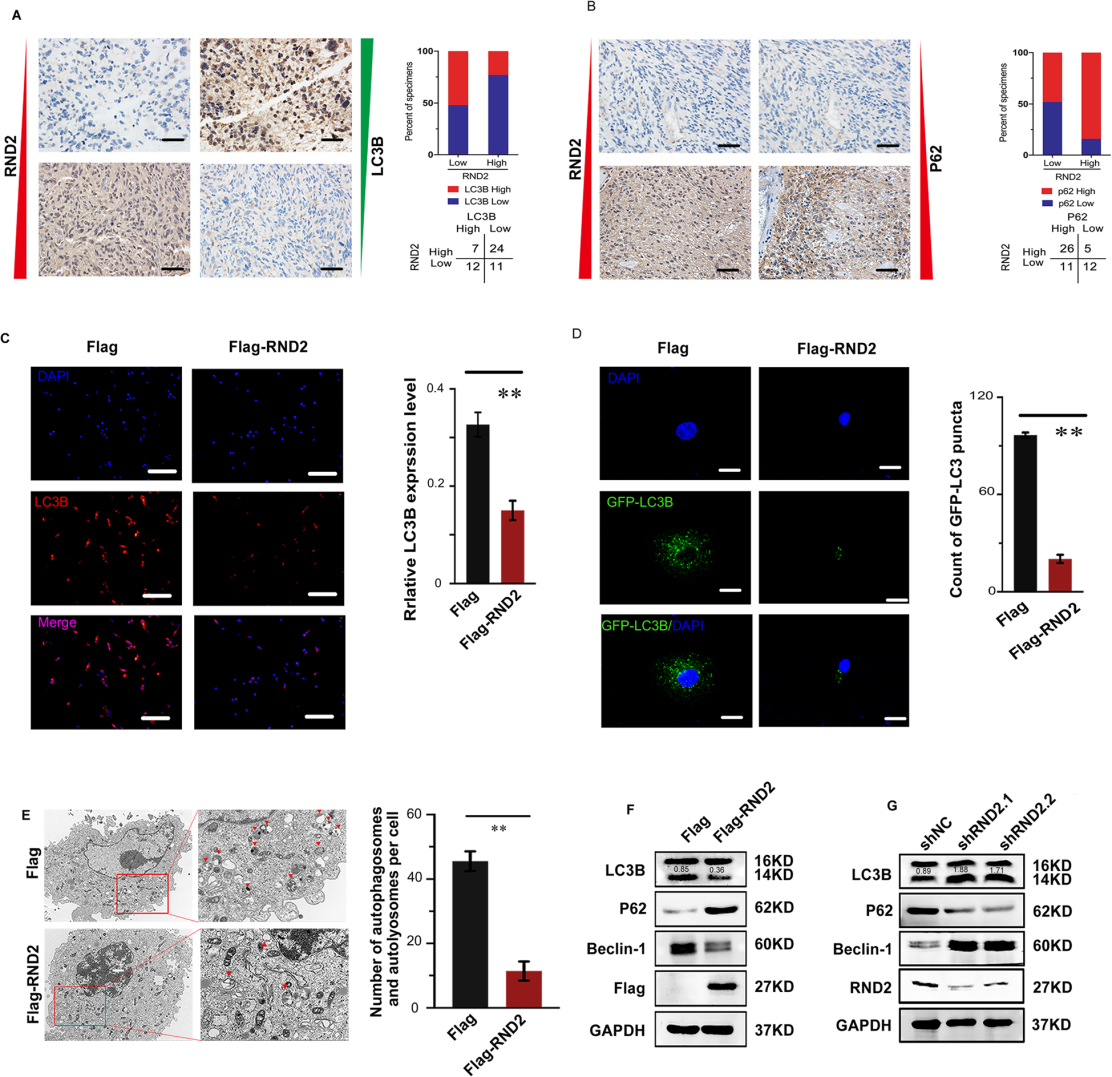
RND2 overexpression reduces GBM cell autophagy in vitro. **a-b** Representatives images of IHC staining showed RND2 expression inversely correlates with LC3B in the same location but positively correlates with p62. Scale bars, 50 μm. **c** Effects of RND2 overexpression on LC3B expression in U87 cells according to immunofluorescence. Scale bars, 100 μm. **P < 0.01. **d** The numbers of GFP-LC3 puncta were quantified using confocal laser scanning microscopy in U87-MG cells transfected with GFP-LC3. Scale bars, 10 μm. **P < 0.01. **e** Transfected cells were prepared for transmission electron microscopy analysis. The number of autophagic vacuoles was analysed and the red arrows indicate autophagic vacuoles. **f** Effects of RND2 overexpression on the levels of autophagy-related proteins in GBM cells. **g** Effects of RND2 knockdown on the levels of autophagy-related proteins in GBM cells. **P < 0.01. shNC: negative control shRNA; shRND2–1 and shRND2–2: two shRNAs against RND2; Flag: the control group; Flag-RND2: RND2 overexpression group. All bar plot data are the means ± SD. The data and graphs are representative of three independent experiments with similar results

Because autophagy is one of the most important mechanisms of cell death, further, we wanted to explore whether RND2 played a key role in regulating autophagy in glioblastoma cells. First, in the same location of human glioblastoma samples we found by immunochemistry staining that RND2 was negatively correlated with LC3B (Fig. 3a) but positively correlated with p62 (Fig. 3b), which are both markers of autophagy [20]. Furthermore, we found that the level of RND2 mRNA was negatively correlated with LC3B but positively correlated with p62 by RT-PCR (Fig. S3A, B). In summary, these data suggested that RND2 had a negative relationship with autophagy.

Further, we detected the effect of RND2 in glioblastoma cells by up- or downregulating its expression in U87 and U251 cells. Immunofluorescence results for LC3B showed that the cells overexpressing RND2 had lower fluorescence intensities (Fig. 3c). Additionally, we stably expressed GFP-LC3B in U87 cells to facilitate the visualization of autophagy, and we found that the overexpression of RND2 inhibited GFP-LC3 puncta formation compared with control cells (Fig. 3d). In addition, we also showed that the number of autophagic vacuoles per cell was evidently lower in the RND2 overexpression group compared with the control group (Fig. 3e). Moreover, RND2 overexpression decreased LC3B and Beclin-1 levels but increased p62 levels (Fig. 3f). Inversely, these proteins were expressed at opposite levels when RND2 was knocked down (Fig. 3g). In a summary, RND2 could inhibit autophagic cell death.

### RND2 overexpression reduced cell apoptosis and autophagy in an intracranial Xenograft model

**Fig. 4:**
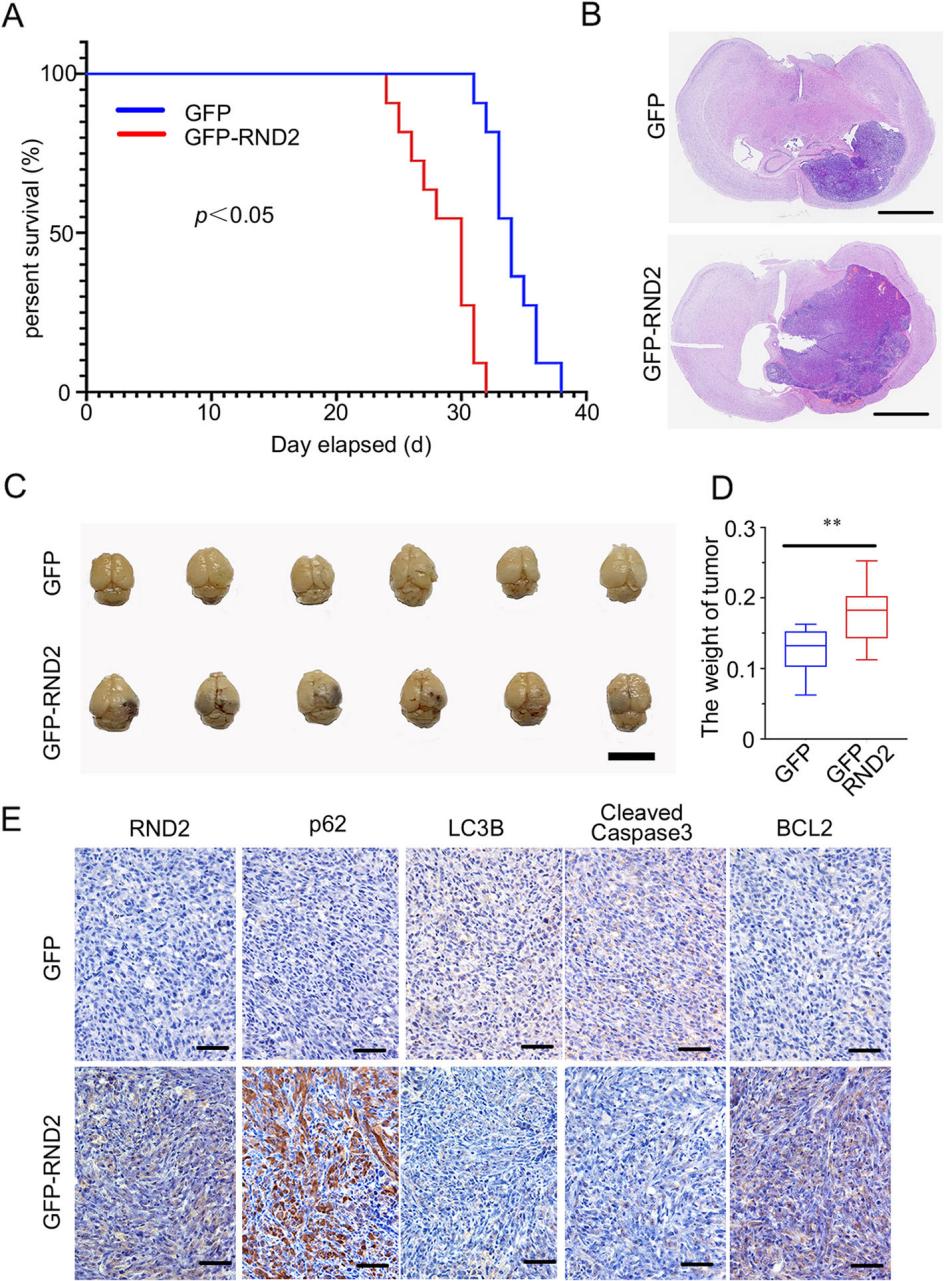
RND2 overexpression attenuates autophagy and apoptosis in an Intracranial Xenograft Model. **a** Mouse survival is shown by Kaplan-Meier curves. GFP groups, *n* = 12; GFP-RND2 groups, *n* = 12. *P < 0.05. *P* values were calculated using the log-rank test. **b** Representative images of mouse brain sections and quantification of tumour volumes from mice that were intracranially implanted with GFP and GFP-RND2 with indicated modifications. Scale bars, 1.0 mm. **c** Representative images of mouse brains. **d** The weight of tumours (GFP/GFP RND2 group – PBS group) were analysed. **P < 0.01. **e** IHC analyses of RND2, p62, LC3B, cleaved caspase-3 and Bcl-2 in mouse tumour sections

We established a stable overexpression of RND2 in U87 cells and constructed an intracranial xenograft model for the purpose of investigating the potential effects of RND2 in vivo. First, we tested the efficacy of RND2 overexpression, which was ensured by western blot and RT-PCR (Fig. S6A, B). Next, we created an intracranial xenograft model by implanting U87 cells intracranially. According to the Kaplan-Meier curves, mice in the control group survived significantly longer than those in the RND2 overexpression group (Fig. 4a). As expected, the mice that were implanted with the RND2 overexpression U87 cells had larger tumours than the mice in the control group (Fig. 4b, c). Additionally, the weights of the tumours in the RND2 overexpression group were significantly greater than those of the control group (Fig. 4d). Immuno-histochemical assays showed that RND2 led to higher expression levels of p62 and Bcl-2 and lower expression levels of cleaved caspase-3 and LC3B (Fig. 4e). These results also indicated that RND2 could weaken autophagy and apoptosis in vivo.

### RND2 physically interacted with p38 and inhibited p38 phosphorylation

Notch signalling, NF-kb signalling, p53 signalling and Snail1 signalling also play key roles in glioblastoma cell death [21–23]. We next explored the signalling activity of these pathways by measuring target gene expression by RT-PCR and western blot. Our results showed that there were no significant differences when RND2 was overexpressed (Fig. S4).

**Fig. 5:**
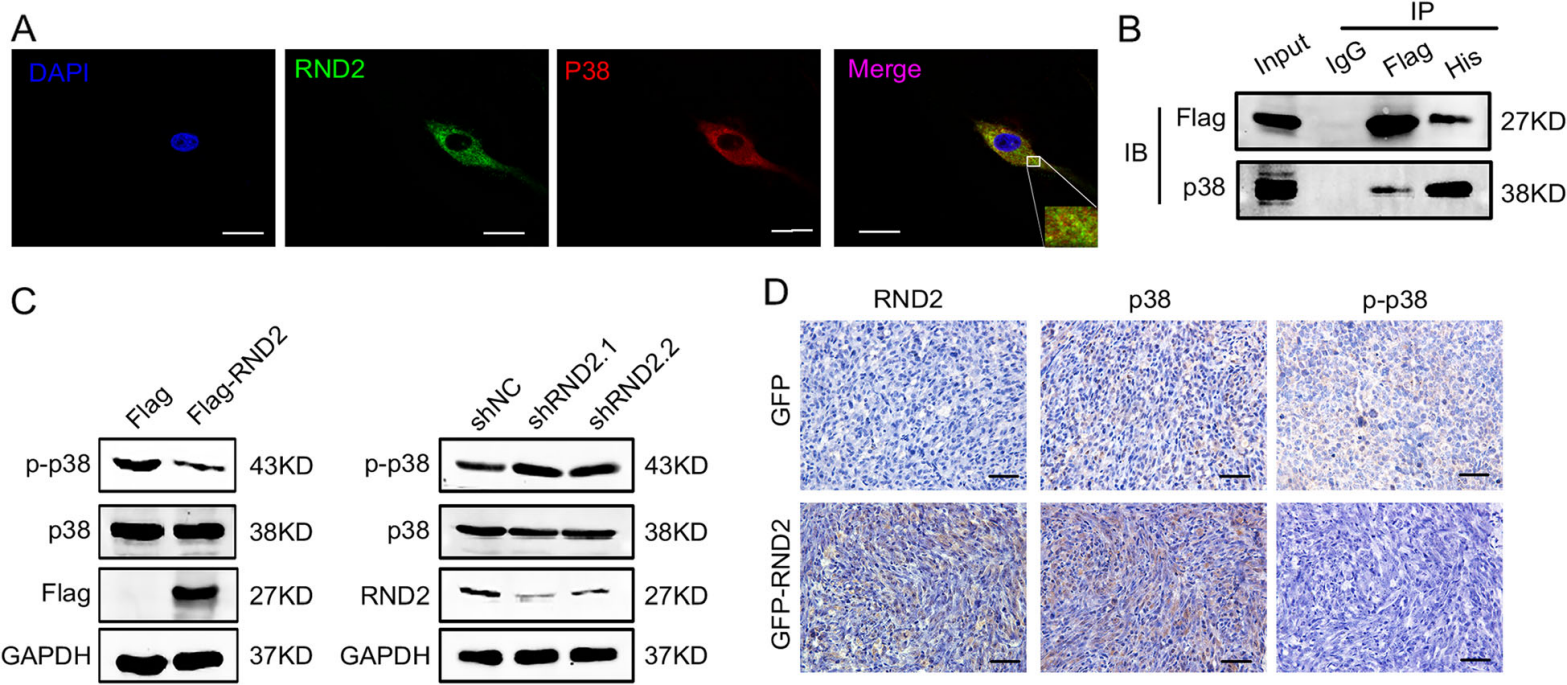
RND2 physically interacts with p38 and inhibits p38 phosphorylation in vitro and in vivo. **a** Immunofluorescent staining (IF) analyses of the co-localization of exogenous RND2 and p38 in U87 cells, Scale bar 10 μm. **b** Co-immunoprecipitation assays in U87 cells determined the physical interaction between RND2 and p38. **c** Effects of RND2 overexpression and knockdown on the protein levels of p-p38 and p38 in U87 cells. **d** IHC analyses of RND2, p-p38 and p38 in mouse tumour sections. shNC: negative control shRNA; shRND2–1 and shRND2–2: two shRNAs against RND2; pcDNA3.1: the control group; Flag-RND2: RND2 overexpression group. All bar plot data are the means ± SD. The data and graphs are representative of three independent experiments with similar results

It has also been reported in previous studies that the activated p38 MAPK signalling pathway can induce cell death in cancer [6]. To determine whether RND2 regulated the p38 MAPK signalling pathway, we detected p-p38 and p38 levels. We found that p38 levels were not influenced by RND2, however, p-p38 was reduced when RND2 was overexpressed and increased when RND2 was knocked down in U87 and U251 cells (Fig. 5c and Fig. S5G). Consistently, p-p38 was reduced when RND2 was overexpressed in vivo (Fig.5d). It is known that p38 phosphorylation is an indicator of p38 MAPK signal activation [5]; therefore, these results indicate that RND2 can reduce p38 MAPK signal activation in glioblastoma cells.

We used co-immunoprecipitation and immunofluorescence assays, which could demonstrate the association between proteins, to explore the potential mechanism of how RND2 decreases p-p38 expression levels. First, the subcellular localization of endogenous RND2 in GBM cells was examined by immunofluorescence, and the results showed that the endogenous RND2 was expressed not only in the cytoplasm but also in cellular membranes (Fig. S5E). Furthermore, the co-localization of RND2 and p38 in GBM patient tissues was observed by immunofluorescence mainly in the cytoplasm (Fig. S5A). Consistent with these results, co-immunoprecipitation assays in U87 cells demonstrated the physical interaction between RND2 and p38 in U87 and U251 cells (Fig. 5b and Fig. S5D). In U87 cells, p38 was distributed mainly in the cytoplasm but was detected in the nucleus as well, however, p38 was found to be co-localized with RND2 only in the cytoplasm (Fig. 5a and Fig. S5B, C), which was in accordance with our previous results. Notably, the overexpression of RND2 resulted in a huge decrease in the level of nuclear p-p38 (Fig. S5F). Further, we found that RND2 was negatively correlated with p-p38 expression in clinical patient samples (Fig. S3C, D). In summary, these results indicated that the activity of the p38 MAPK signalling pathway was downregulated by RND2 and that p38 regulates its own downstream target genome.

### p38 induced autophagy in GBM cells and rescued RND2-mediated autophagy and apoptosis

**Fig. 6:**
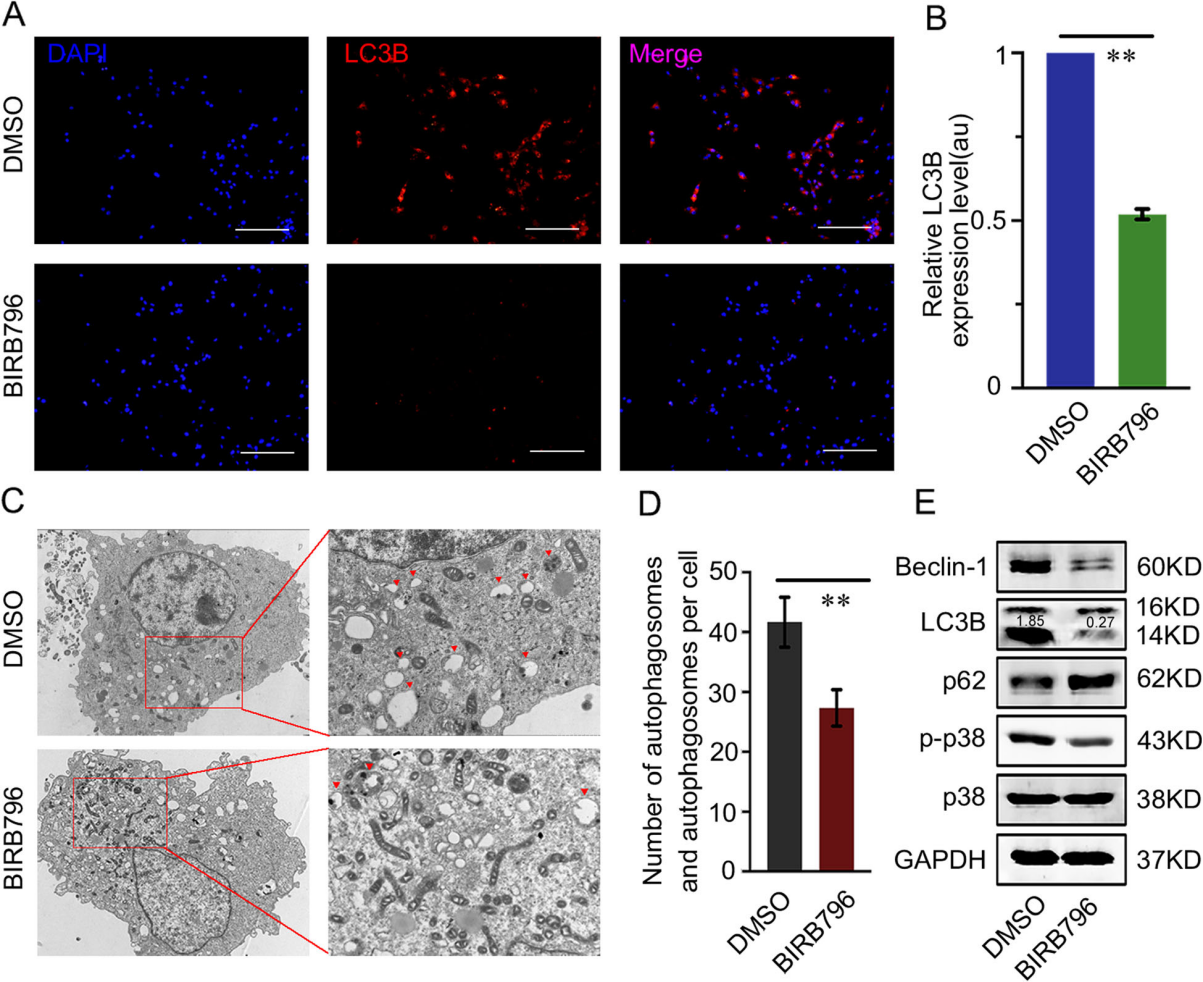
Inhibiting p38 phosphorylation reduced autophagy in vitro. **a**-**b** Effects of p38 de-phosphorylation on LC3B expression in U87 cells according to immunofluorescence. Scale bars, 100 μm. **P < 0.01. **c-d** Cells were prepared for transmission electron microscopy analysis. The number of autophagic vacuoles was analysed and the red arrows indicate autophagic vacuoles. **P < 0.01. **e** Effects of p38 de-phosphorylation on the levels of autophagy-related proteins in U87 cells

The p38 MAPK signalling pathway can regulate autophagy and cell death, however, its regulation of autophagy responses can be both positive and negative [14, 24]. Therefore, in the next experiments, we explored the role of p38 MAPK signalling in autophagy of GBM cells. BIRB796, an inhibitor of p38 phosphorylation, was used block the activity of p38 MAPK signalling. The results showed that cells had lower fluorescence intensity when they were treated with BIRB796 (Fig. 6a, b). We also showed that the number of autophagic vacuoles per cell was evidently lower in the BIRB796 group than the DMSO group (Fig. 6c, d). The western blot results showed that BIRB796 decreased LC3B and Beclin-1 levels but increased p62 levels (Fig. 6e). All of these data demonstrated that p38 MAPK signalling increased autophagy in human glioblastoma cells.

**Fig. 7:**
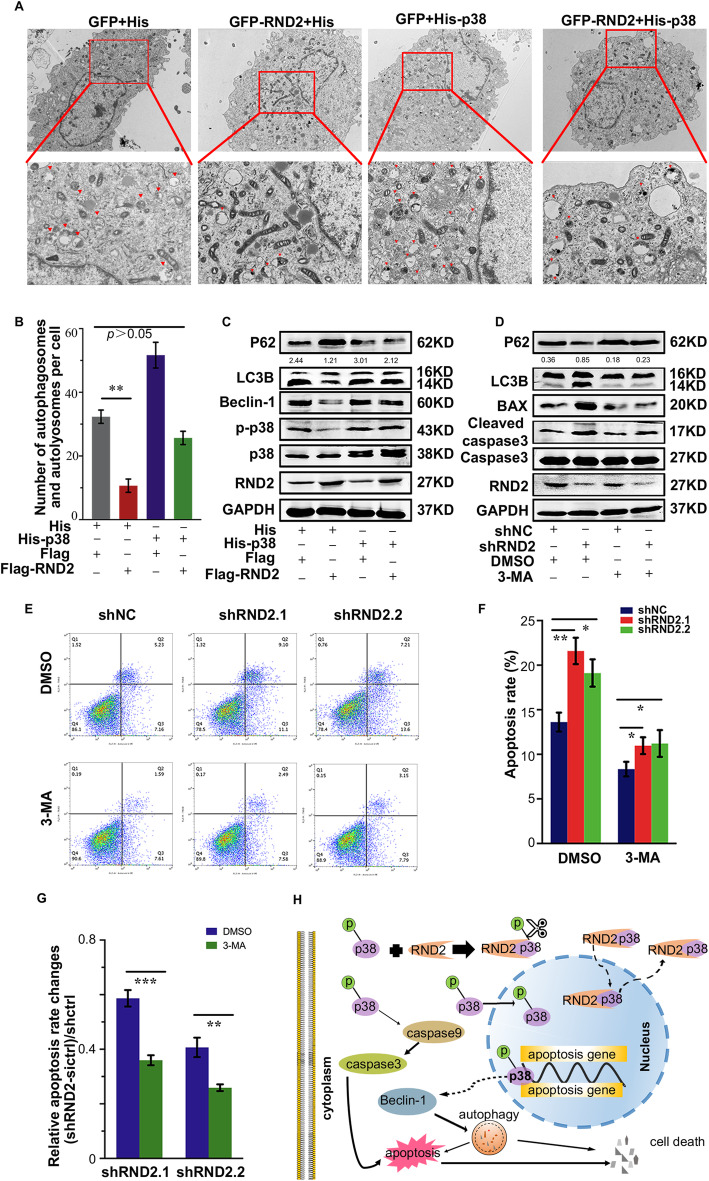
RND2 attenuates autophagy by inhibiting p38 MAPK signalling pathway activation. **a-b** p38 rescued cell autophagy induced by RND2 expression. Cells were prepared for transmission electron microscopy analysis. The number of autophagic vacuoles was analysed and the red arrows indicate autophagic vacuoles. **P < 0.01. **c** Overexpressing p38 rescued the inhibition of autophagy-related proteins caused by RND2 in U87 cells. **d** 3-MA decreases the levels apoptosis-related protein caused by RND2. **e-g** 3-MA downregulated RND2-mediated apoptosis in U87MG cells, with the relative apoptosis rate significantly downregulated by 3-MA. Cell apoptosis was determined by FACS. **p < 0.01, ***p < 0.001. **h** Mechanistic model for RND2 regulation of cell autophagy and apoptosis in GBM

As demonstrated previously, RND2 was able to target the p38 MAPK signalling pathway in GBM cells. Furthermore, we speculated that p38 could be involved in RND2-mediated autophagy and apoptosis. The average number of autophagic vacuoles in one cell was calculated using TEM, and we found that the number soared in U87 cells with overexpressed p38, which could rescue the inhibitory effects of RND2 (Fig. 7a, b). Furthermore, we detected autophagy-mediated protein levels and found that overexpressing p38 could rescue the inhibition of autophagy caused by RND2 (Fig. 7c, Fig. S2E). Additionally, 3-MA, an inhibitor of autophagy, decreased apoptosis caused by knockdown of RND2 in U87-MG (Fig. 7e, f), and the relative apoptosis rate was significantly decreased in the 3-MA group compared to the DMSO group (Fig. 7g). Additionally, western blot results revealed that the effect of 3-MA on autophagy was significantly decreased during RND2-mediated GBM cell apoptosis (Fig. 7d, Fig. S2F).

In summary, all of our results demonstrated that RND2 could inhibit autophagy and decrease apoptosis by inhibiting the p38 MAPK signalling pathway. Furthermore, RND2-associated autophagy inhibition is a component of GBM cell apoptosis resistance.

### RND2 predicted the poor prognosis of patients suffering from GBM

**Fig. 8:**
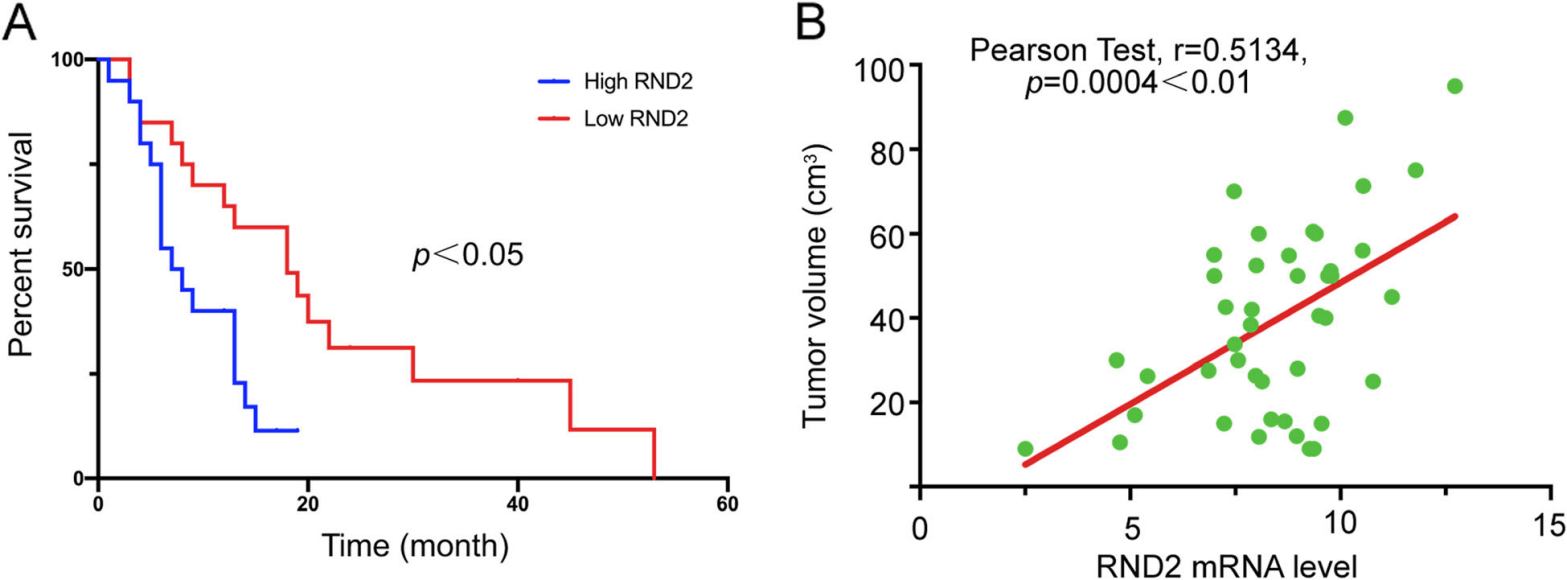
RND2 Predicted Poor Prognosis of Patients with GBM. **a** Kaplan-Meier analyses for GBM patients with high or low level of RND2 expression in tumours. *p < 0.05*. **b** Correlation between RND2 expression level and tumour volume in clinical GBM patients

To further evaluate the role of RND2 in clinical samples, we performed RT-PCR assays to examine the levels of RND2 expression, as shown above (Fig. 1f), in tumour tissues sampled from patients suffering from primary GBM. In this experiment, we identified low and high RND2 expression using the RND2 mRNA expression levels and carried out a Kaplan-Meier analysis (Table 1); all relevant information is mentioned in Table S2. Kaplan-Meier curves showed that the patients in the low RND2 expression group survived significantly longer than the patients in the high expression group (Fig. 8a). Additionally, RND2 expression was positively correlated with tumour volume (Fig. 8b).

**Table 1:** Kaplan-Meier analysis of 41 clinical GBM patients

Clinicopathologic variables	Number	Average survival time	***P*** value
All cases	41	16.475 ± 2.600	
Age at diagnosis (years)			0.382
<55	19	12.888 ± 1.908	
≥ 55	22	20.189 ± 4.422	
Gender			0.618
Male	22	18.556 ± 4.335	
Female	19	14.289 ± 2.814	
KPS			0.736
≤ 70	8	15.000 ± 4.979	
>70	33	17.698 ± 3.306	
Headache			0.904
Yes	18	12.752 ± 1.692	
No	23	17.195 ± 3.676	
Intracranial infection			0.052
Yes	3	6.000 ± 4.041	
No	38	17.352 ± 2.759	
Multiple lesions			0.746
1	33	16.708 ± 2.740	
≥ 2	8	10.250 ± 1.980	
Volume			0.192
≥ 42	20	12.075 ± 2.216	
<42	21	19.473 ± 4.138	
Lobe lesions			0.584
1	33	15.996 ± 2.907	
≥ 2	8	17.469 ± 4.948	
Course time(d)			0.151
≥ 30	14	10.399 ± 1.738	
<30	27	18.820 ± 3.488	
RND2 expression			
High	20	9.307 ± 1.189	0.017*
Low	21	21.296 ± 3.981

In summary, these data revealed that RND2 could be defined as a biomarker for glioblastomas and may indicate poor prognosis in GBM patients.

## Discussion

Our results showed for the first time that the RND2/p38/MAPK signalling axis regulates cell death including autophagic activities and apoptosis in GBM. Upregulated RND2 expression in GBM was defined as a negative predictor in patients. Constitutively expressed or induced RND2 decreased the phosphorylation of p38 through physical interaction, thereby inhibiting GBM cell autophagy and apoptosis (Fig. 7h).

GBM is the most frequently seen malignant primary tumour in the central nervous system of adults. Even with ever-accelerating treatments, including radical surgery, radiotherapy and chemotherapy, the overall survival time of patients suffering from GBM only remains at approximately 18 months [1]. The evasion of and resistance to apoptosis are hallmarks of malignant tumours [25], which suggests that apoptosis may be a therapeutic strategy for anti-tumour drugs. Additionally, GBM cells lack the intrinsic apoptosis pathway, which leads to chemo-resistance and treatment failure [26, 27]. Furthermore, autophagy is identified as type II programmed cell death, especially in cells with apoptosis deficiencies [28]. However, other studies have shown that autophagy can inhibit the development of GBM in the early stage but promote the survival of GBM cells in the late stage. In recent years, clinical studies of autophagy inhibitors in glioblastoma have not yielded the expected results, which indicates that the role autophagy plays in cell death is complicated [29]. Consequently, it is necessary to explore more specific targets that regulate autophagy to inhibit the development of GBM. Hence, how to induce glioblastoma cell death by autophagy and apoptosis is an urgent problem to solve and is significant for clinical treatment.

RND2 is a member of the RND subfamily, which is a subfamily of the Rho GTPases. The main feature of RND2 is its lack of intrinsic and GAP-stimulated GTPase activities [30]. In addition, its function does not rely on GDP/GTP exchange but rather on transcriptional, post-translational, and post-transcriptional mechanisms [31]. The activities of RND2 have been explored not only in normal tissue development but also in disease states [10]. However, the activities of RND2 in cancer have not yet been demonstrated. Additionally, the mechanistic and direct role of RND2 in GBM tumour genesis is totally unexplored. To date, only a few proteins including Rapostlin, MgcRacGAP and Vps4-A have been identified as RND2 binding partners [12, 32, 33]. Our study is the first to advance the knowledge of RND2 in GBM and cell death. Our data showed that the level of RND2 expression was dramatically decreased in GBM compared with normal brain tissues. Besides, the expression level of RND2 was higher in PN and CL compared with MES. As we all know, MES subtype is generally considered to hint worse prognosis, however, it’s showed that the favourable outcome of the proneural GBM subtype was because patients were IDH mutant and when those patients are excluded from analysis, the proneural subtype has a worse prognosis than other subtypes [34]. The PN group expresses genes associated with the process of neurogenesis [35] and RND2 plays an important role in the development of brain [10]. This may explain that the expression of RND2 is higher in PN compared with MES. Furthermore, RND2 was negatively correlated with patient prognosis, while it was positively correlated with tumour size, suggesting that RND2 is a potential target for treating GBM.

Our study firstly identified and validated that p38 was the substrate for RND2. Besides, we found that RND2 decreased the phosphorylation of p38 by directly binding to p38 in the cytoplasm of cancer cells and we observed that RND2-mediated p38 MAPK signalling was critical for autophagy activities. The activity of p38 MAPK was related to the enhanced expression of autophagic markers (such as ATG5/ATG12 and LC3B) and apoptotic markers (such as PARP and caspase-3) [36, 37]; thus, p38 MAPK is quite critical in regulating the balance between survival and cell death [5]. As we know, there are many downstream substrates that could be regulated by p38 mitogen-activated protein kinase, which regulates various cellular processes through a cascade of complex phosphorylation [38]. p38 signalling suppresses tumourigenesis and promotes apoptosis in various types of cancers. Consequently, it is important to understand the mechanisms of how specific substrates are recognized and regulated by p38. There may be some factors, such as availability, concentration, and subcellular localization of upstream proteins [39], that influence substrate selection. It has been found that p38 is expressed in both the cytoplasm and the nucleus (but mainly in the cytoplasm), which demonstrates that the distribution of p38 might be critical for regulating substrates. Hence, the potential binding partners of p38 like MK2, MKK3, TAB1 may regulate p38 in different ways such as through phosphorylation sites or subcellular localization [40–42]. It is possible RND2 associates with these proteins complex and mediates p38 nucleus-cytoplasm transportation directly or indirectly.

Our study further found that RND2/p38/MAPK signalling axis downregulates cell death, owing to the fact that the inhibition of autophagy coincided with the inhibition of apoptosis- and autophagy-mediated cell death, with the inhibition of autophagy further attenuating apoptosis. However, p38 played a dual role in autophagy. For example, the phosphorylation of p38 could promote the expression of the key autophagy protein Beclin-1, leading to cell death [14]. Inversely, p38 MAPK inhibited autophagy by phosphorylating ULK1 [24].

Our data provided significant evidence that RND2 inhibited autophagy through the de-phosphorylation of p38 specifically. Autophagy has significant functions under different pathological conditions but crosstalk with apoptosis is still controversial [43]. It has been pointed out that autophagy occurs before apoptosis and that it is a necessary condition. Autophagy suppresses the development of tumours by inhibiting the expression of oncogenes and promoting pro-apoptotic factors to induce the cell death [44]. However, chemo-resistance is one of the most common problems during anti-cancer therapy and autophagy is closely related to this process through its involvement in the avoidance of chemotherapy-induced apoptosis in different cancers. Moreover, autophagy provides nutrients for cells in a hypoxic or starvation environment to protect tumour cells from apoptosis in GBM [45]. In addition, autophagy and apoptosis were found to be antagonistic or synergistic under certain conditions. Apoptosis and autophagy could occur simultaneously to trigger cell death, while apoptosis could also accelerate the transformation of cells to autophagic cell death [46, 47]. Above all, our study revealed that the RND2/p38/MAPK signalling axis downregulated autophagy and apoptosis at the same time and that apoptosis could be influenced by RND2-mediated autophagy to improve the survival of GBM cells. Our results enrich our knowledge of the mechanism by which autophagy is precisely regulated in glioblastomas, which may provide a potential solution for TMZ chemo-resistance to TMZ in GBM cells which have emerged as a challenging problem in clinical practice [48]. Lack of programmed cell death is an important reason which caused chemo-resistance. Recently, it has been demonstrated that autophagy plays a prodeath role in GBM cells treated with chemotherapeutic agents, by enhancing autophagy-mediated apoptosis [49]. Similarly, our results show that RND2 promote the survival of cells by reducing autophagy and autophagy-mediated apoptosis in GBM. In a summary, our data not only suggests RND2 as an alternative therapeutic target for malignant human cancers such as GBM but also provides a solid foundation for the development of a compound targeting RND2, which could be transformed into clinical applications and combined with chemotherapies.

## Conclusion

In summary, our study showed that RND2 regulates the p38 MAPK pathway along with cell death in GBM. RND2 binds to p38 directly and inhibits the phosphorylation of the p38 protein, leading to the inhibition of the p38 MAPK signalling pathway, which downregulated apoptosis and autophagy in GBM cells. In clinical GBM samples, RND2 was defined as an oncogene that predicted a poor clinical outcome in patients. These new discoveries help us to comprehend the regulation of the p38 MAPK signalling pathway in GBM. As a result, RND2 could be a potential therapeutic target for use in the treatment of GBM.

## Supplementary information

**Additional file 1: Figure S1.** The expression of RND2 in normal tissue. (A) Expression of RND2 in normal human tissues according to the HPA database. (B-C) mRNA and protein levels of RND2 from different organs of C57 mice. (D-E) Immunofluorescence staining showed the relationship between RND2, GFAP, and NSE. Scale bar, 50 μm.

**Additional file 2: Figure S2.** RND2 influenced apoptosis and autophagy and p38 rescued RND2-mediated apoptosis and autophagy in U251 cells. (A-B) U251 cells were transfected with negative control shRNA (shNC) or shRNAs against RND2 (shRND2–1 and shRND2–2), followed by Annexin V-PE/7-AAD staining and flow cytometric analysis. Cell apoptosis was determined by FACS. **P < 0.01, *P < 0.05. (C) Effects of RND2 overexpression and knockdown on the levels of apoptosis-related proteins in U251 cells. (D) Effects of RND2 overexpression and knockdown on the levels of autophagy-related proteins in U251 cells. (E) Overexpressing p38 rescued the inhibition of autophagy-related proteins caused by RND2 in U251 cells. (F) 3-MA downregulated RND2-mediated apoptosis in U251 cells, with the relative apoptosis rate significantly downregulated by 3-MA. shNC: negative control shRNA; shRND2–1 and shRND2–2: two shRNAs against RND2; pcDNA3.1: the control group; Flag-RND2: RND2 overexpression group. All bar plot data are the means ± SD. The data and graphs are representative of three independent experiments with similar results.

**Additional file 3: Figure S3.** RND2 inhibited autophagy in clinical samples. (A-B) Correlation between RND2 mRNA levels and tumour volume in clinical GBM patients. (C-D) Correlations of IHC data for high or low RND2 expression relative to the level of p-p38.

**Additional file 4: Figure S4.** RND2 overexpression did not regulate key proteins in GBM. (A-G) Hes1, IL-8, KRas, MMP-2, P21, Slug and Smad4 mRNA levels when RND2 was overexpressed. (H) STAT3, Snail-1, p53, p65, and HES1 protein levels when RND2 was overexpressed.

**Additional file 5: Figure S5.** RND2 physically interacts with p38 and inhibits p38 phosphorylation in vitro. (A) The co-localization of RND2 and p38 in GBM patient tissues was observed by immunofluorescence. Scale bar, 50 μm. (B-C) Statistical descriptions of RND2 and p38 co-localization in U87 cells. (D) Co-immunoprecipitation assays in U251 cells determined the physical interaction between RND2 and p38 in U251 cells. (E) Endogenous RND2 was expressed not only in the cytoplasm but also in cellular membranes. (F) Effects of RND2 on p-p38 expression in U87 cells according to immunofluorescence. Scale bars, 10 μm. (G) Effects of RND2 overexpression and knockdown on the protein levels of p-p38 and p38 in U251 cells.

**Additional file 6: Figure S6.** Detecting RND2 in cells stably overexpression RND2. (A, B) Efficacy of RND2 overexpression; the process was ensured by western blot and RT-PCR.

**Additional file 7.**

**Additional file 8.**

**Additional file 9.**

## Data Availability

The data used to support the findings of this study are available from the corresponding author upon request.

## Abbreviations

CL: Classical
GBM: Glioblastoma multiform
MAPK: Mitogen-activated protein kinase
IHC: Immunohistochemistry
IDH1/2: Isocitrate dehydrogenase 1/2
MES: Mesenchymal
TEM: Transmission Electron Microscopy
TUNEL: TdT-mediated dUTP Nick-End Labeling
PN: Proneural
