# Supplementary figures and images for: RND2 attenuates apoptosis and autophagy in glioblastoma cells by targeting the p38 MAPK signalling pathway
Source: J Exp Clin Cancer Res. 2020 Aug 31;39:174. doi: 10.1186/s13046-020-01671-2 (PMC7457501; doi:10.1186/s13046-020-01671-2)

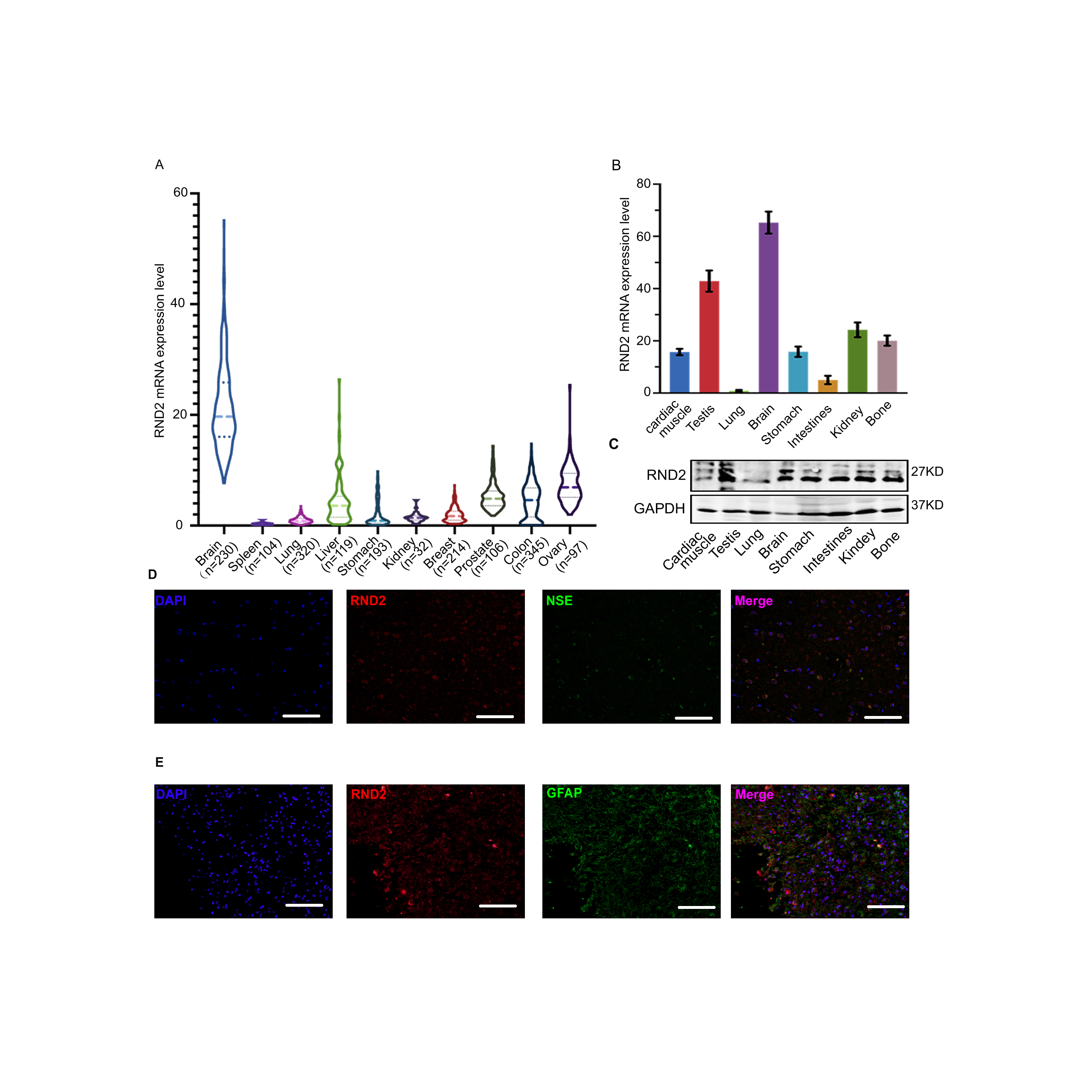

Supplement: Supplementary file 1 — Additional file 1: Figure S1. The expression of RND2 in normal tissue. (A) Expression of RND2 in normal human tissues according to the HPA database. (B-C) mRNA and protein levels of RND2 from different organs of C57 mice. (D-E) Immunofluorescence staining showed the relationship between RND2, GFAP, and NSE. Scale bar, 50 μm. [file 13046_2020_1671_MOESM1_ESM.png]

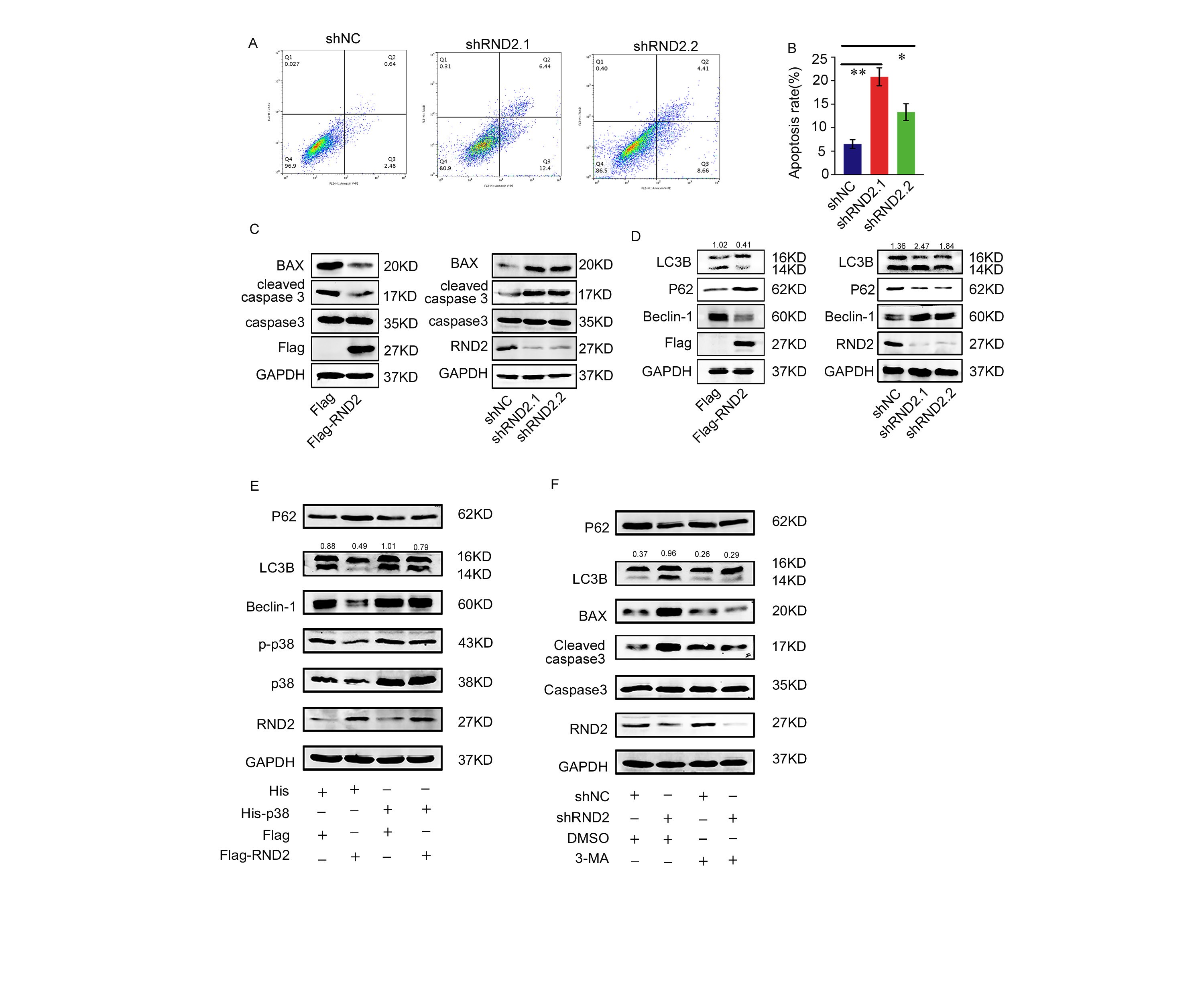

Supplement: Supplementary file 2 — Additional file 2: Figure S2. RND2 influenced apoptosis and autophagy and p38 rescued RND2-mediated apoptosis and autophagy in U251 cells. (A-B) U251 cells were transfected with negative control shRNA (shNC) or shRNAs against RND2 (shRND2–1 and shRND2–2), followed by Annexin V-PE/7-AAD staining and flow cytometric analysis. Cell apoptosis was determined by FACS. **P < 0.01, *P < 0.05. (C) Effects of RND2 overexpression and knockdown on the levels of apoptosis-related proteins in U251 cells. (D) Effects of RND2 overexpression and knockdown on the levels of autophagy-related proteins in U251 cells. (E) Overexpressing p38 rescued the inhibition of autophagy-related proteins caused by RND2 in U251 cells. (F) 3-MA downregulated RND2-mediated apoptosis in U251 cells, with the relative apoptosis rate significantly downregulated by 3-MA. shNC: negative control shRNA; shRND2–1 and shRND2–2: two shRNAs against RND2; pcDNA3.1: the control group; Flag-RND2: RND2 overexpression group. All bar plot data are the means ± SD. The data and graphs are representative of three independent experiments with similar results. [file 13046_2020_1671_MOESM2_ESM.png]

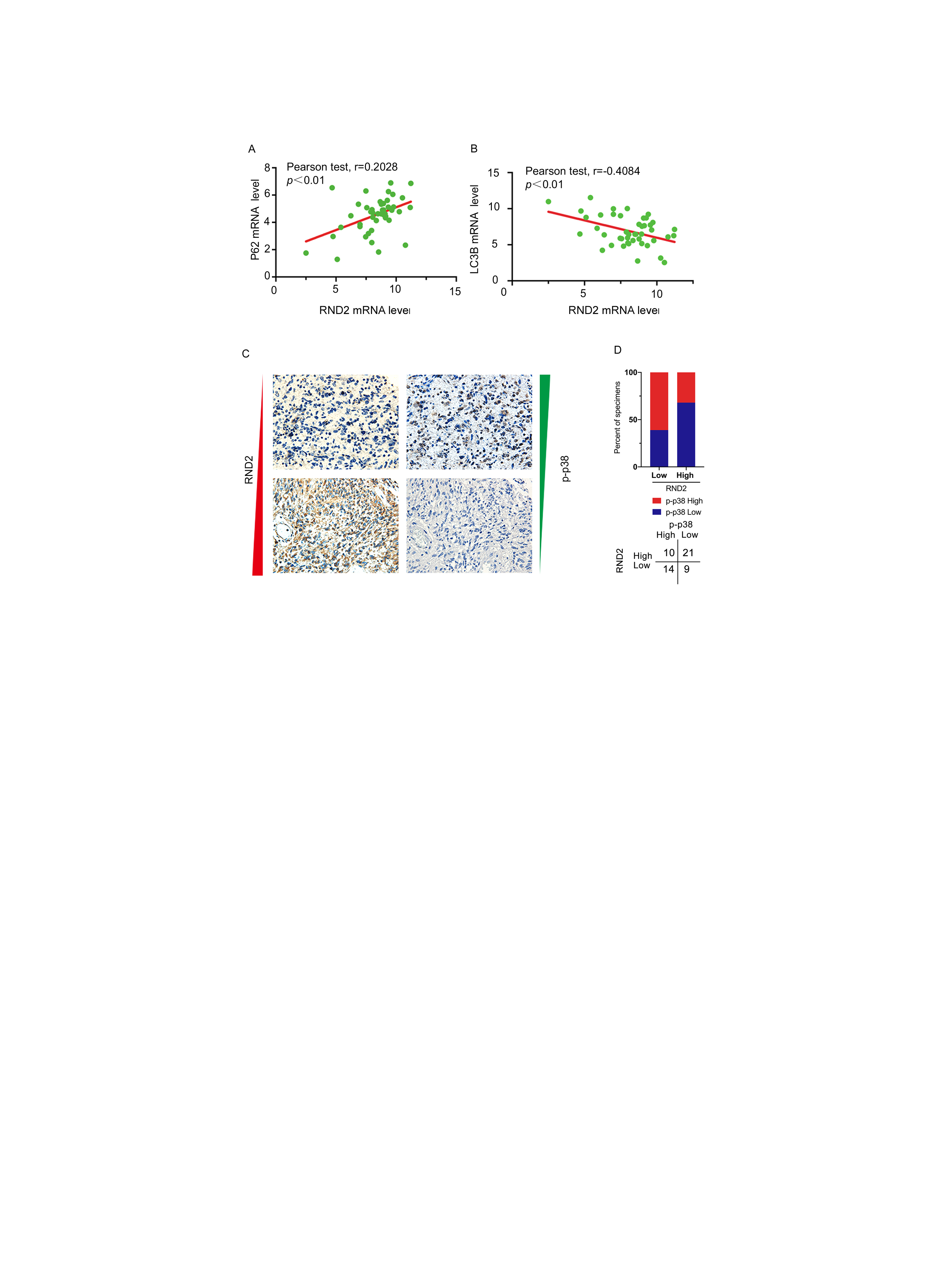

Supplement: Supplementary file 3 — Additional file 3: Figure S3. RND2 inhibited autophagy in clinical samples. (A-B) Correlation between RND2 mRNA levels and tumour volume in clinical GBM patients. (C-D) Correlations of IHC data for high or low RND2 expression relative to the level of p-p38. [file 13046_2020_1671_MOESM3_ESM.png]

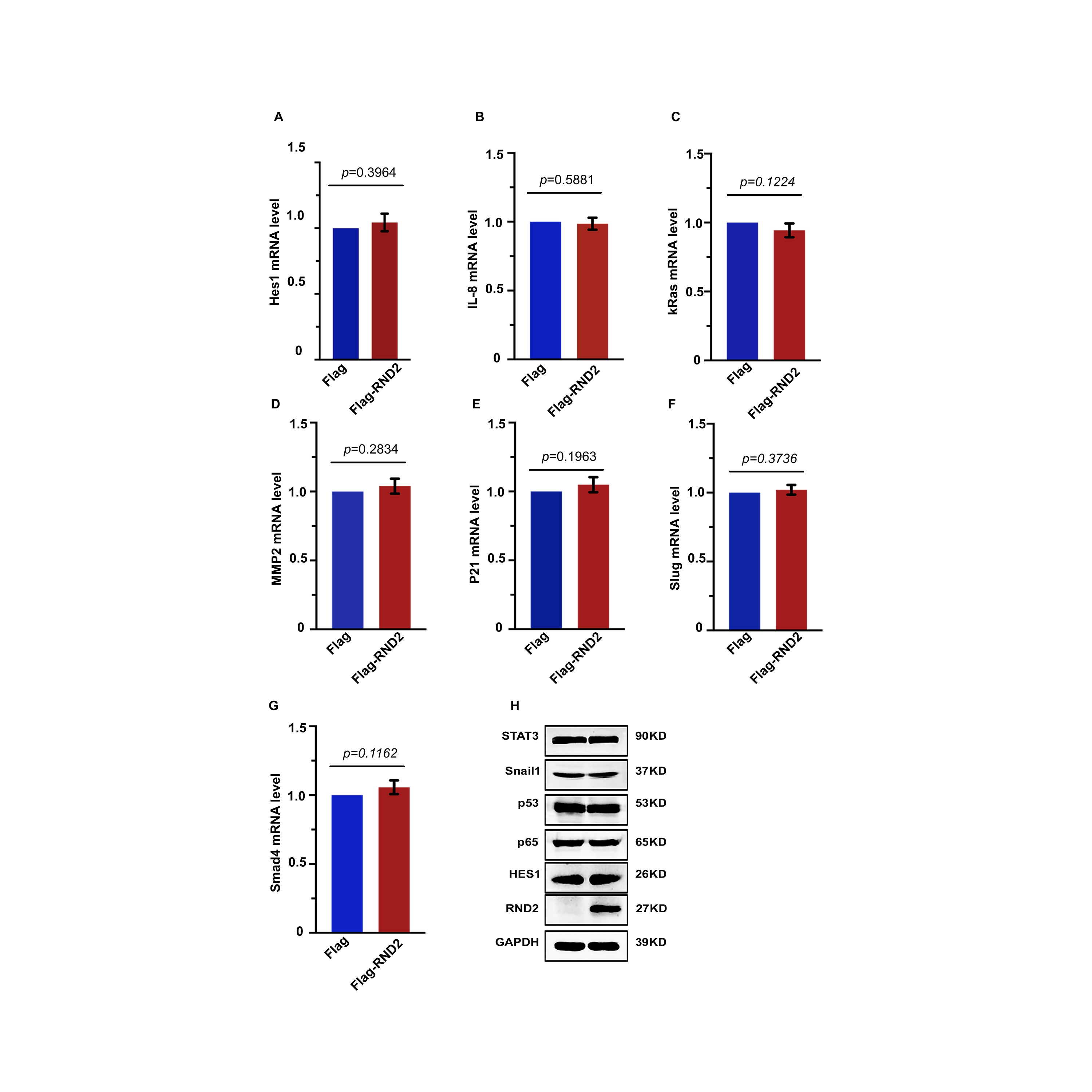

Supplement: Supplementary file 4 — Additional file 4: Figure S4. RND2 overexpression did not regulate key proteins in GBM. (A-G) Hes1, IL-8, KRas, MMP-2, P21, Slug and Smad4 mRNA levels when RND2 was overexpressed. (H) STAT3, Snail-1, p53, p65, and HES1 protein levels when RND2 was overexpressed. [file 13046_2020_1671_MOESM4_ESM.png]

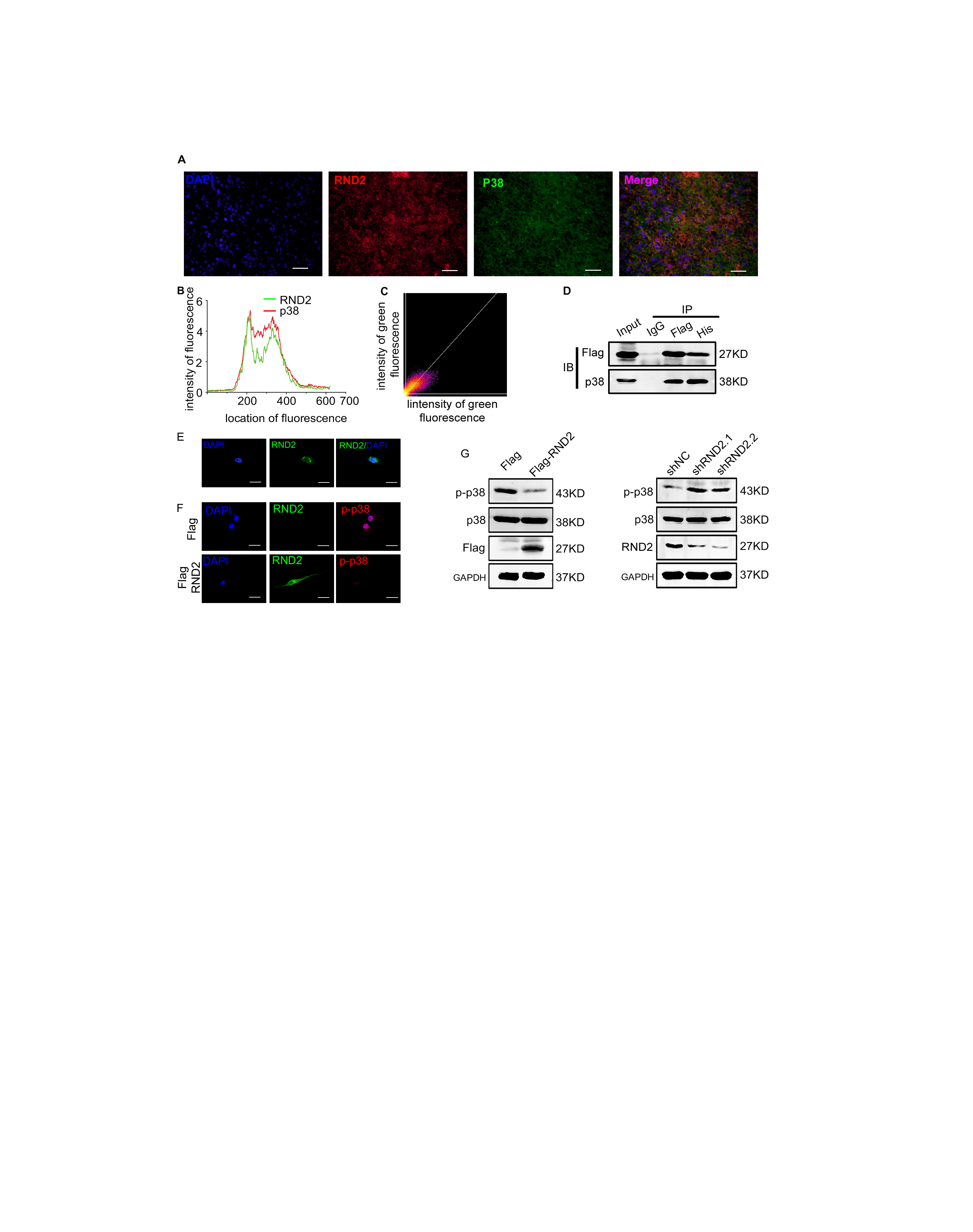

Supplement: Supplementary file 5 — Additional file 5: Figure S5. RND2 physically interacts with p38 and inhibits p38 phosphorylation in vitro. (A) The co-localization of RND2 and p38 in GBM patient tissues was observed by immunofluorescence. Scale bar, 50 μm. (B-C) Statistical descriptions of RND2 and p38 co-localization in U87 cells. (D) Co-immunoprecipitation assays in U251 cells determined the physical interaction between RND2 and p38 in U251 cells. (E) Endogenous RND2 was expressed not only in the cytoplasm but also in cellular membranes. (F) Effects of RND2 on p-p38 expression in U87 cells according to immunofluorescence. Scale bars, 10 μm. (G) Effects of RND2 overexpression and knockdown on the protein levels of p-p38 and p38 in U251 cells. [file 13046_2020_1671_MOESM5_ESM.png]

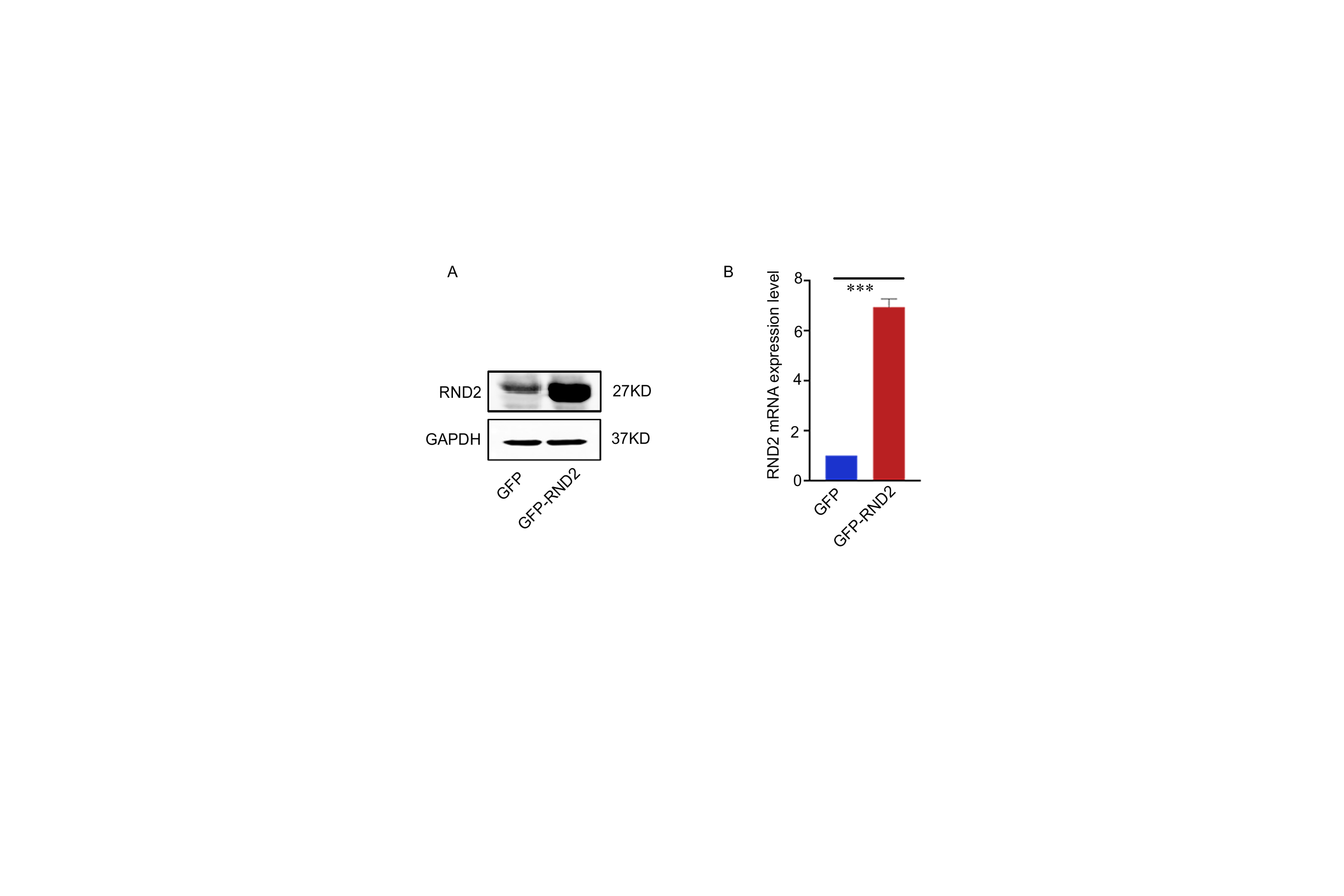

Supplement: Supplementary file 6 — Additional file 6: Figure S6. Detecting RND2 in cells stably overexpression RND2. (A, B) Efficacy of RND2 overexpression; the process was ensured by western blot and RT-PCR. [file 13046_2020_1671_MOESM6_ESM.png]
